# Supplementary material for: Longitudinal Metabolomics Data Analysis Informed by Mechanistic Models
Source: Metabolites. 2024 Dec 24;15(1):2. doi: 10.3390/metabo15010002 (PMC11766892; doi:10.3390/metabo15010002)
Supplement: Supplementary file 1 [file metabolites-15-00002-s001.zip › metabolites-3332549-supplementary.pdf]

Supplementary File

A Females vs. Males: Comparison of CP and ACMTF models

In order to understand the performance difference in males vs. females, we compare the factors extracted by CP and ACMTF models. Fig S.1 shows that CP and ACMTF models extract more similar subject scores in $\mathbf{a}_2$ (the component showing statistically significant group difference in terms of BMI) in females compared to males. The factor match score (FMS) between CP and ACMTF factors (taking into account all three components) is also higher for females (FMS=0.70) than males (FMS=0.63).

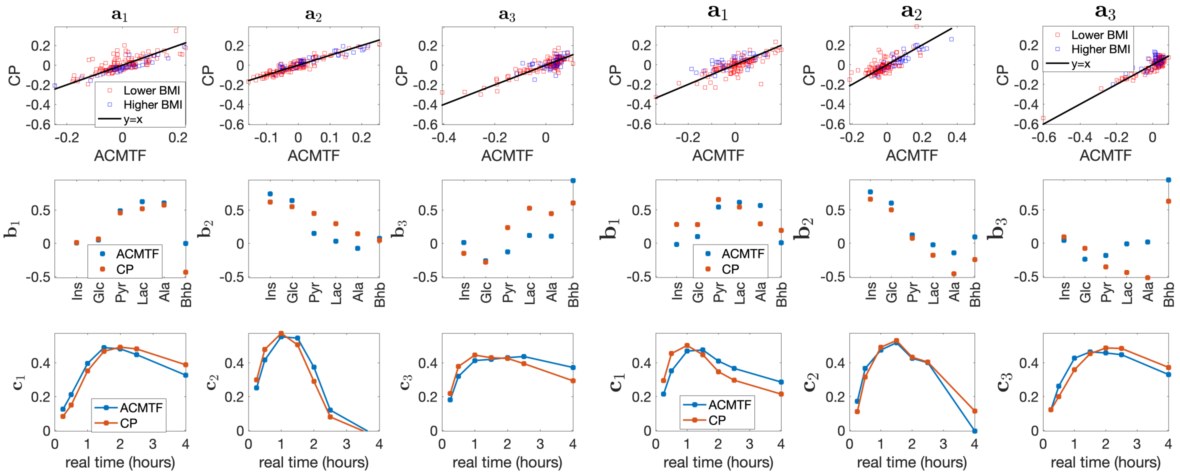

(a) Females (b) Males

Figure S.1: Factors extracted by 3-component CP and ACMTF models: (a) Females, (b) Males.

B Males: Selection of the number of components and regularization parameter

B.1 CP model of real metabolomics data

CP models (without any regularization) using 2, 5, and 6 components are degenerate. For more details on model degeneracy, see [1]. Therefore, we consider 3- and 4-component CP models and assess their replicability. Fig S.2a shows that 95% of the FMS values (see the definition of FMS in subsection *Model Selection* in the main text) are greater than 0.87 and 0.58 using the 3-component and 4-component CP models, respectively. Therefore, we select the 3-component CP model.

To determine the regularization parameter, we also assess the replicability of the 3-component CP model using different regularization parameter values (γ). Fig S.2b shows that $\gamma = 0.01$ is a good choice in terms of replicability and model fit.

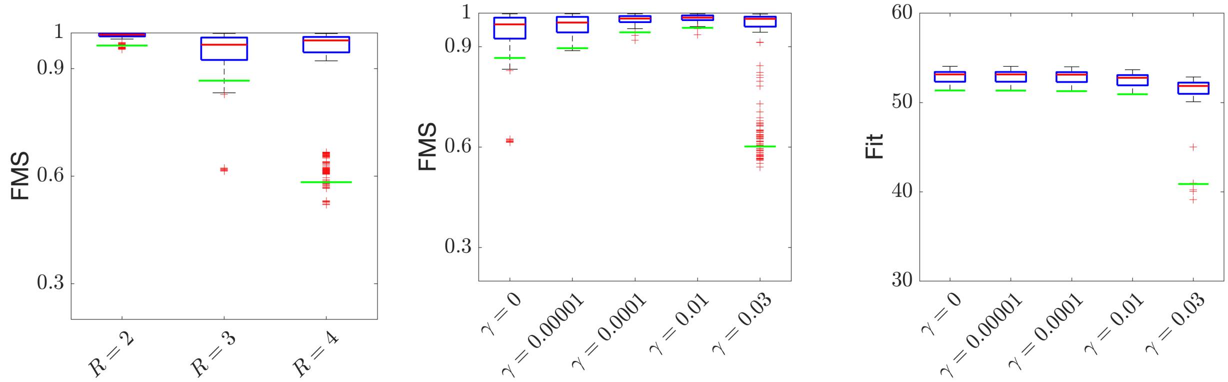

(a) Replicability - no regularization (b) Replicability and model fit using different regularization parameters

Figure S.2: Model selection for the CP model of T0-corrected data from males. (a) Replicability of the CP model without any regularization using different number of components, (b) Replicability and model fit (%) of the 3-component CP model using different regularization parameters. Green lines show that 95% of the FMS and model fit values are above that line.

B.2 ACMTF model of real and simulated metabolomics data

Fig S.3 shows that ACMTF models with 2, 3 and 4 components are replicable. When we use a 4-component model, the first component in the 3-component model (i.e., $\langle \mathbf{a}_1, \mathbf{b}_1, \mathbf{c}_1, \mathbf{d}_1, \mathbf{e}_1 \rangle$ in Fig S.4a) which mainly models Pyr, Lac and Ala splits into two components (i.e., $\langle \mathbf{a}_1, \mathbf{b}_1, \mathbf{c}_1, \mathbf{d}_1, \mathbf{e}_1 \rangle$ and $\langle \mathbf{a}_4, \mathbf{b}_4, \mathbf{c}_4, \mathbf{d}_4, \mathbf{e}_4 \rangle$ in Fig S.4b). Given that Pyr, Lac, and Ala exhibit similar dynamic patterns and they are close in the metabolic pathway [2], they are expected to show up together in a single component. Therefore, we select the 3-component ACMTF model.

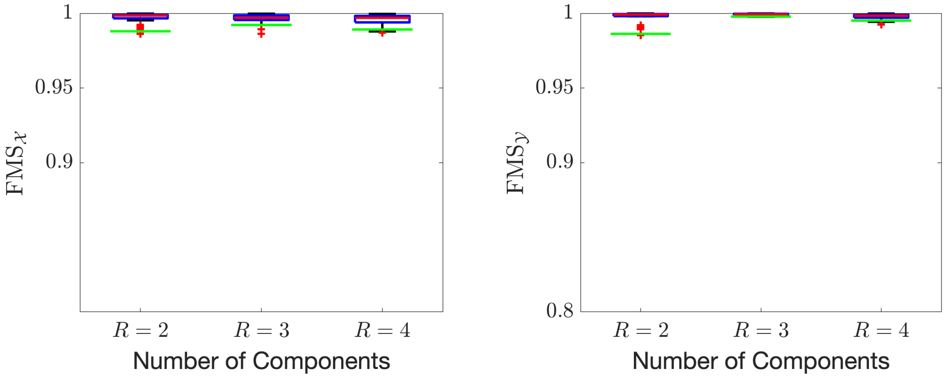

Figure S.3: Replicability of the ACMTF model of T0-corrected real data (from males) and simulated data.

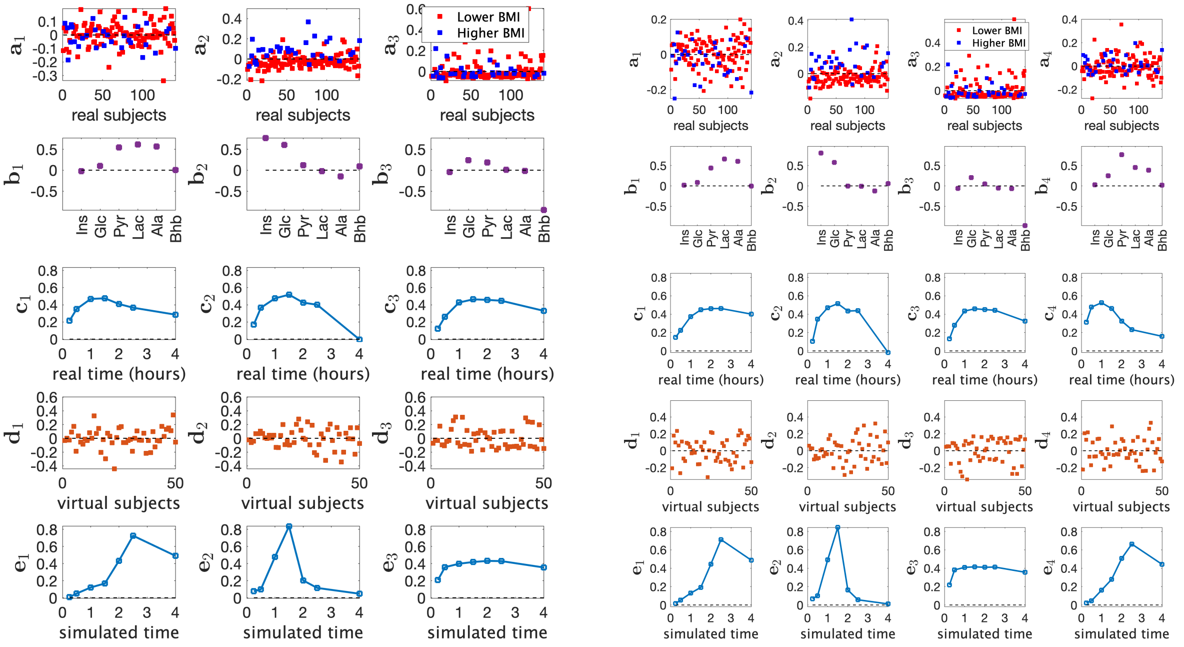

(a) (b)

Figure S.4: 3- and 4-component ACMTF models of T0-corrected real data (from males) and simulated data. $\langle \mathbf{a}_r, \mathbf{b}_r, \mathbf{c}_r, \mathbf{d}_r, \mathbf{e}_r \rangle$, $r = 1, 2, 3, 4$, are the components in the *subjects* (real), *metabolites* (coupled mode), *time* (real), *subjects* (virtual) and *time* (virtual) modes.

C Time profiles of raw data

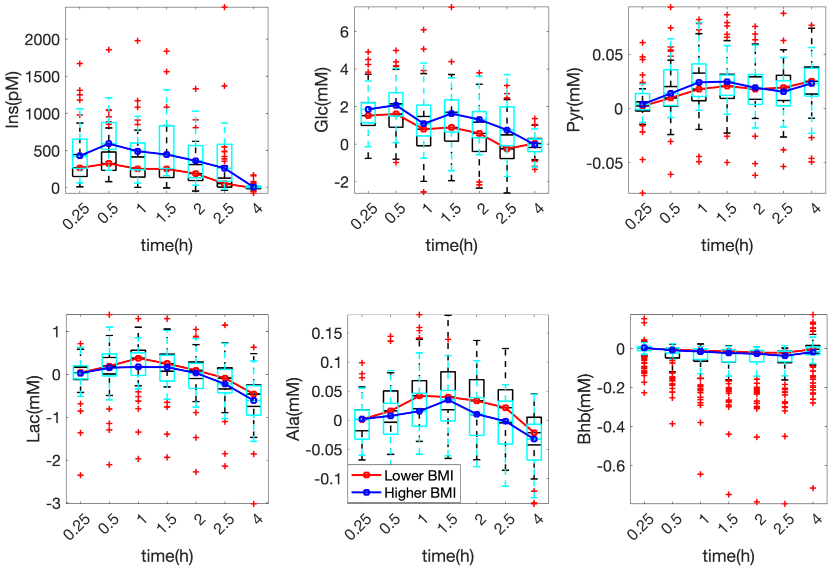

Figure S.5: Median time profiles of 111 male subjects in the *Lower BMI* group vs. 30 male subjects in the *Higher BMI* group based on T0-corrected data.

D Conflicting prior information

D.1 CP model of simulated metabolomics data and construction of simulated data with wrong prior information

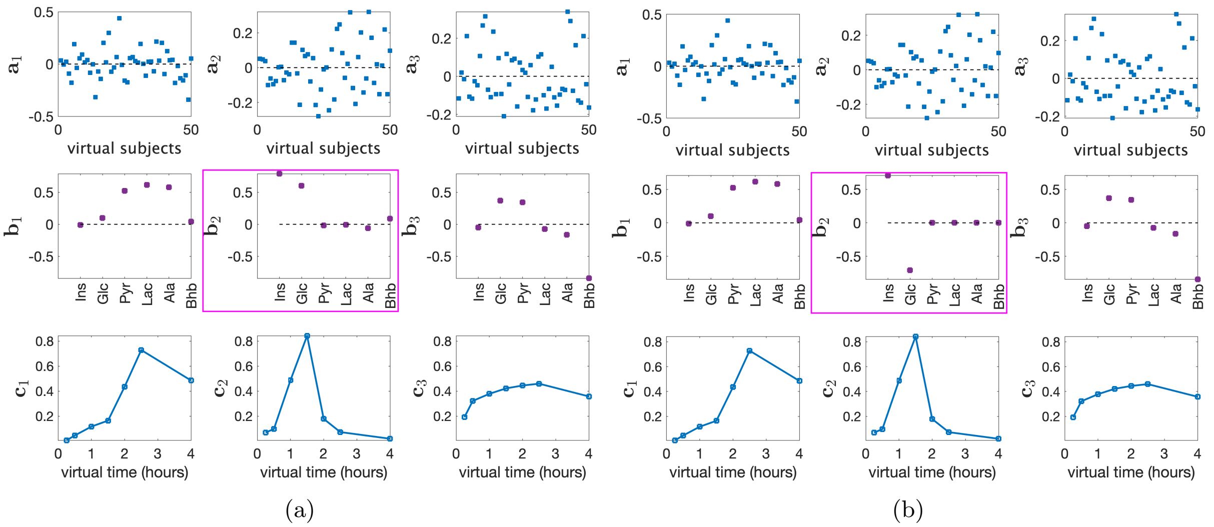

Figure S.6: **(a)** True factors extracted by a 3-component CP model from the default simulated T0-corrected metabolomics data. **(b)** Modified factors used to construct simulated data with wrong prior information. In (a) vs. (b), only $\mathbf{b}_2$ is different. $\mathbf{b}_2$ in Fig S.6b is obtained by setting the values of Ins, Glc, Pyr, Lac, Ala, Bhb to $1, -1, 0, 0, 0, 0$ and then dividing this vector by its 2-norm.

D.2 Joint analysis of real data and simulated data with wrong prior information

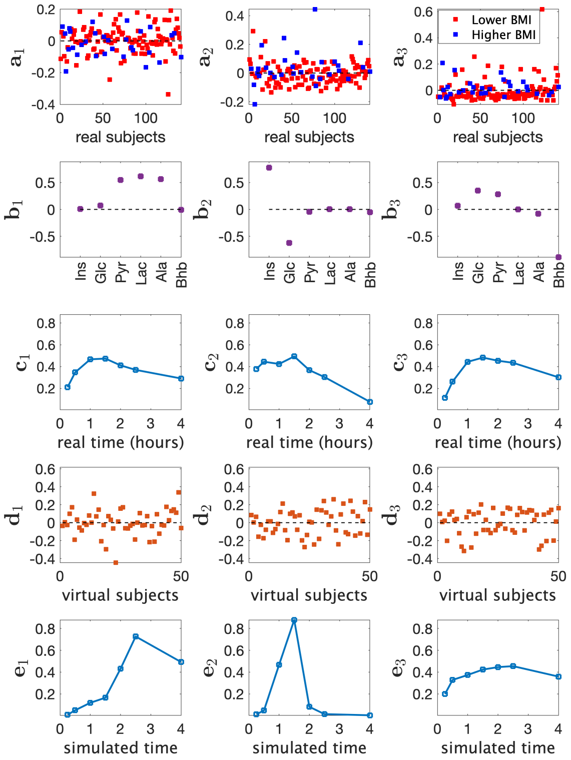

Figure S.7: 3-component ACMTF model of T0-corrected real data from males and simulated data with wrong prior information. $\langle \mathbf{a}_r, \mathbf{b}_r, \mathbf{c}_r, \mathbf{d}_r, \mathbf{e}_r \rangle$, $r = 1, 2, 3$, are the components in the *subjects* (real), *metabolites* (coupled), *time* (real), *subjects* (virtual) and *time* (virtual) modes.

References

[1] T. G. Kolda and B. W. Bader. Tensor decompositions and applications. *SIAM Review*, 51(3):455–500, 2009.

[2] L. Li, S. Yan, B. M. Bakker, H. Hoefsloot, B. Chawes, D. Horner, M. A. Rasmussen, A. K. Smilde, and E. Acar. Analyzing postprandial metabolomics data using multiway models: A simulation study. *BMC Bioinformatics*, 25(94), 2024.
